# Supplementary material for: Dynamic tracking of objects in the macaque dorsomedial frontal cortex
Source: Nat Commun. 2025 Jan 2;16:346. doi: 10.1038/s41467-024-54688-y (PMC11696028; doi:10.1038/s41467-024-54688-y)
Supplement: Supplementary file 1 — Supplementary Information [file 41467_2024_54688_MOESM1_ESM.pdf]

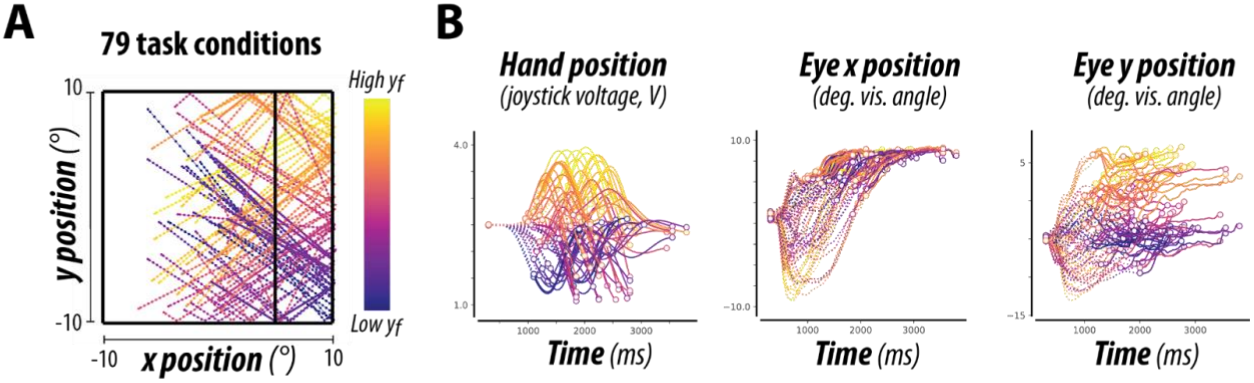

Figure S1. Complementary analysis of behavior. (A) The ball trajectories of the 79 unique conditions used for this study; colors correspond to the per-condition endpoint y-position of the ball (yf). **(B)** Trial-average time-courses of the hand and eye position across all M-Pong conditions, with color mapping identical to panel A (dotted and solid lines for visible and occluded epochs, respectively, with circles to demarcate their boundaries). Source data are provided as a Source Data file.

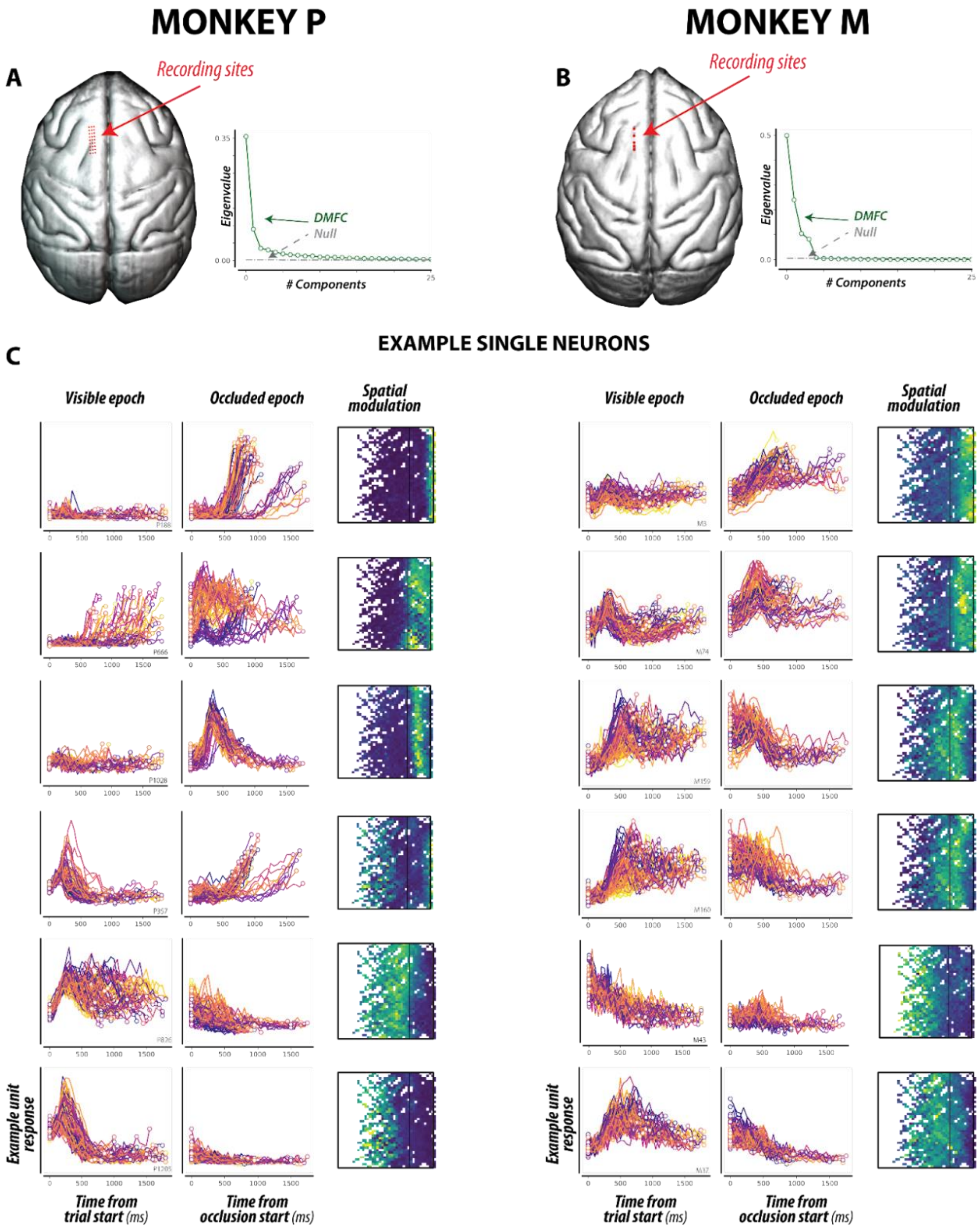

Figure S2. Complementary analyses of single-neuron responses in DMFC. (A,B) Left: Neurophysiology sites in DMFC (red circles) rendered on the cortical surface (extracted from structural MRI) of each monkey. Right: Scree plot showing the dimensionality of neural activity in DMFC for each animal (same format as Fig. 3B. **(C)** Same as Fig. 2C in the main manuscript, shown separately for the two animals (left column: monkey P; right column: monkey M). Source data are provided as a Source Data file.

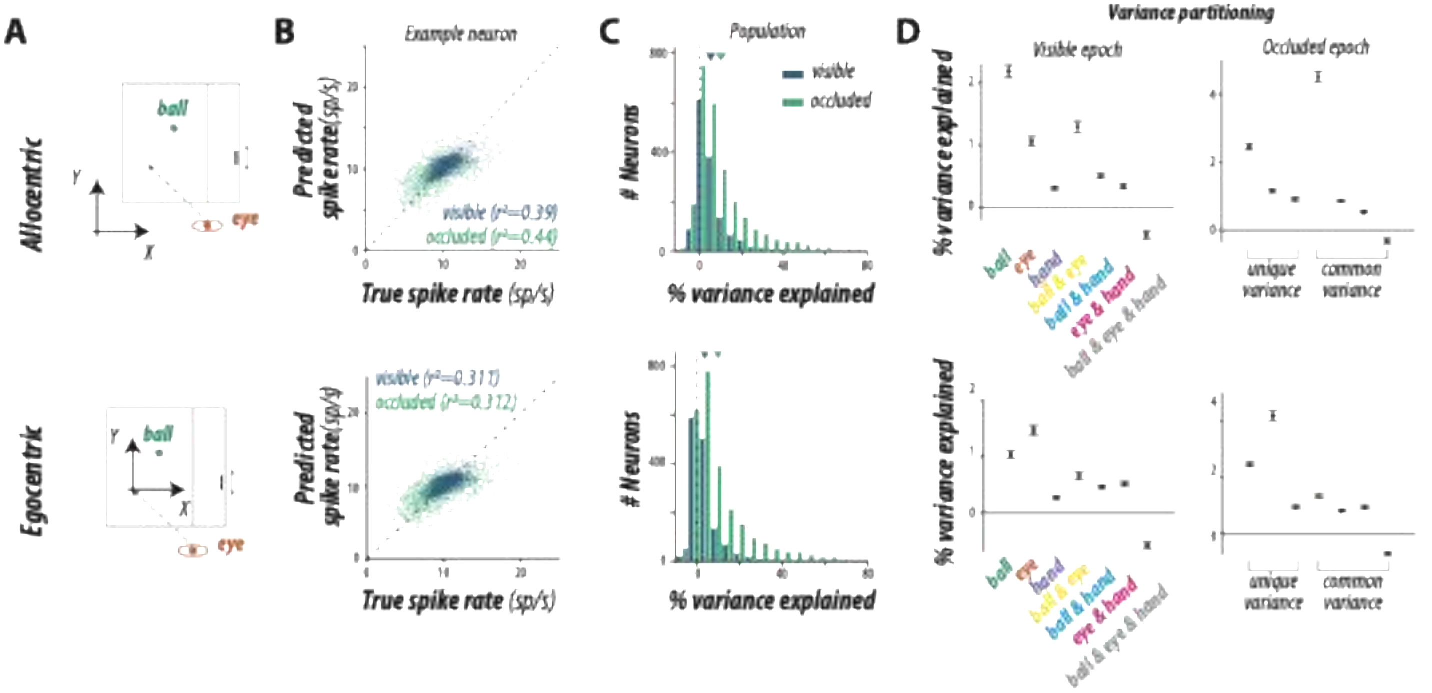

Figure S3. Single-neuron encoding analysis with allocentric versus egocentric framework of reference. (A) (top row) Allocentric model (same as Figure 2D-F). In the main text and Figure 2, we computed the position of the ball in the allocentric frame (i.e., screen coordinate). (bottom row) Egocentric model. We repeated the same series of single-neuron encoding analysis with the ball position coded egocentrically with respect to the eye position (ball x,y position - eye x,y position). The goal of these analyses was to examine how DMFC neurons encode the task-relevant variables such as the positions of the ball, paddle and eye. **(B)** For a representative neuron (top: allocentric, bottom: egocentric), the spike rate predicted from the cross-validated encoding model is shown with the corresponding actual spike rate (blue: visible epoch, green: occluded epoch). **(C)** The histograms (top: allocentric, bottom: egocentric) show the distribution of the percent variance accounted for by the encoding model across neurons (triangles for mean for each epoch). We found no significant difference across neurons between the allocentric and egocentric models (p=0.527, Wilcoxon sign-rank test, N=1385) for visible epoch. For occluded epoch, the difference was significant (p=5.2e-10, Wilcoxon sign-rank test, N=1389). **(D)** Variance partitioning analysis. In the allocentric model (top), the ball-related variables (green) made a strong private contribution to the explained variance in spite of correlations with other motor variables. In the egocentric model (bottom), the ball-associated variances were reduced but still the second highest for both visible and occluded epochs. This finding suggests that DMFC carries strong information about the ball position and speed, regardless of their coding framework. Source data are provided as a Source Data file.

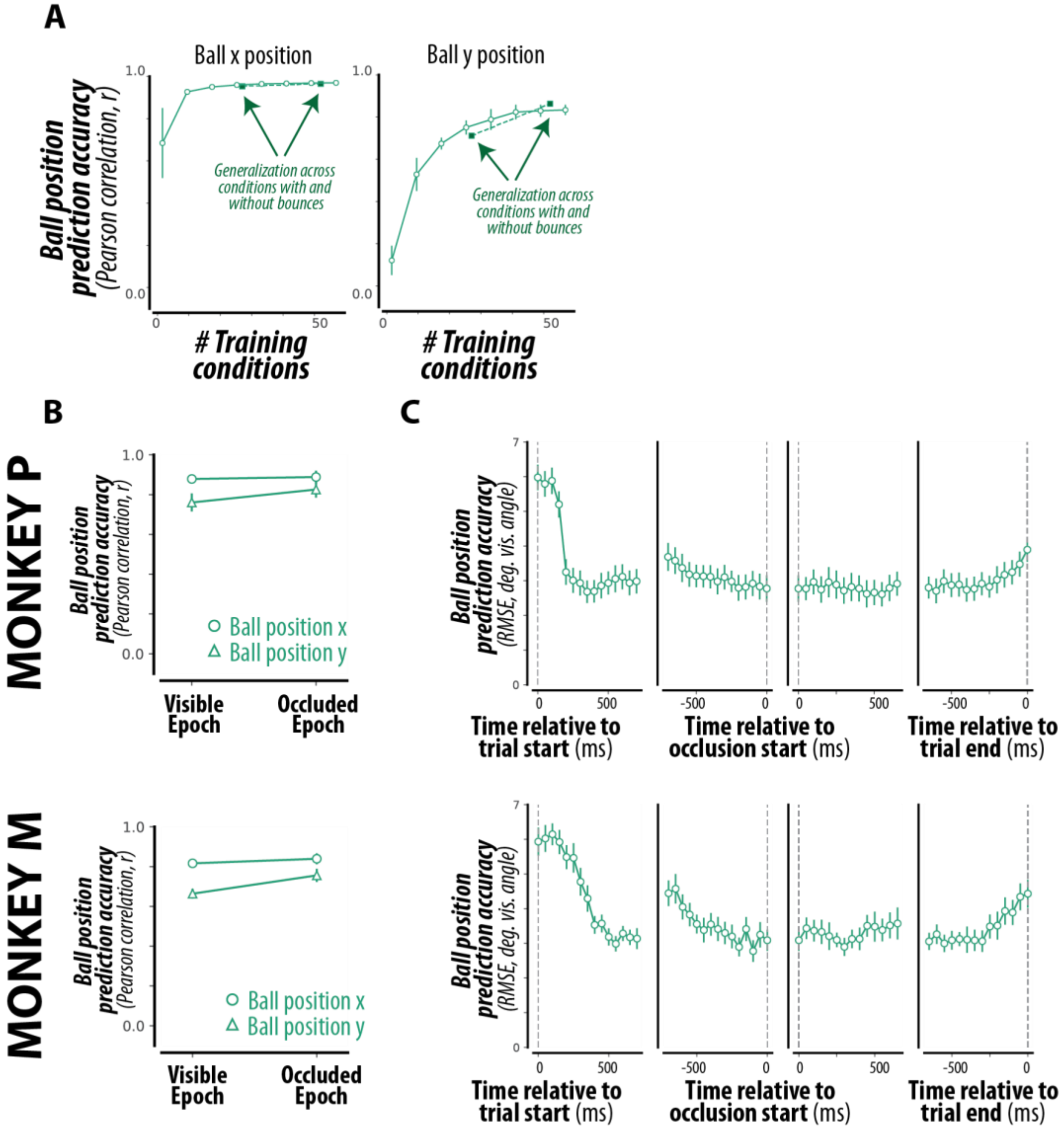

Figure S4. Complementary analyses of the DMFC population responses. (A) Robustness of latent object readout (related to Figure 3E). Accuracy of the readout of ball position x and y (left and right panel, respectively), as a function of the number of Mental-Pong conditions used for training. Read-outs could accurately generalize across conditions from training on a smaller number of conditions. The green squares indicate generalization from training on conditions with bounces and testing on conditions without bounces, and vice versa. Error bars denote mean±SEM. **(B,C)** Same as Fig. 3E,F in the main manuscript, shown separately for the two animals. Source data are provided as a Source Data file.

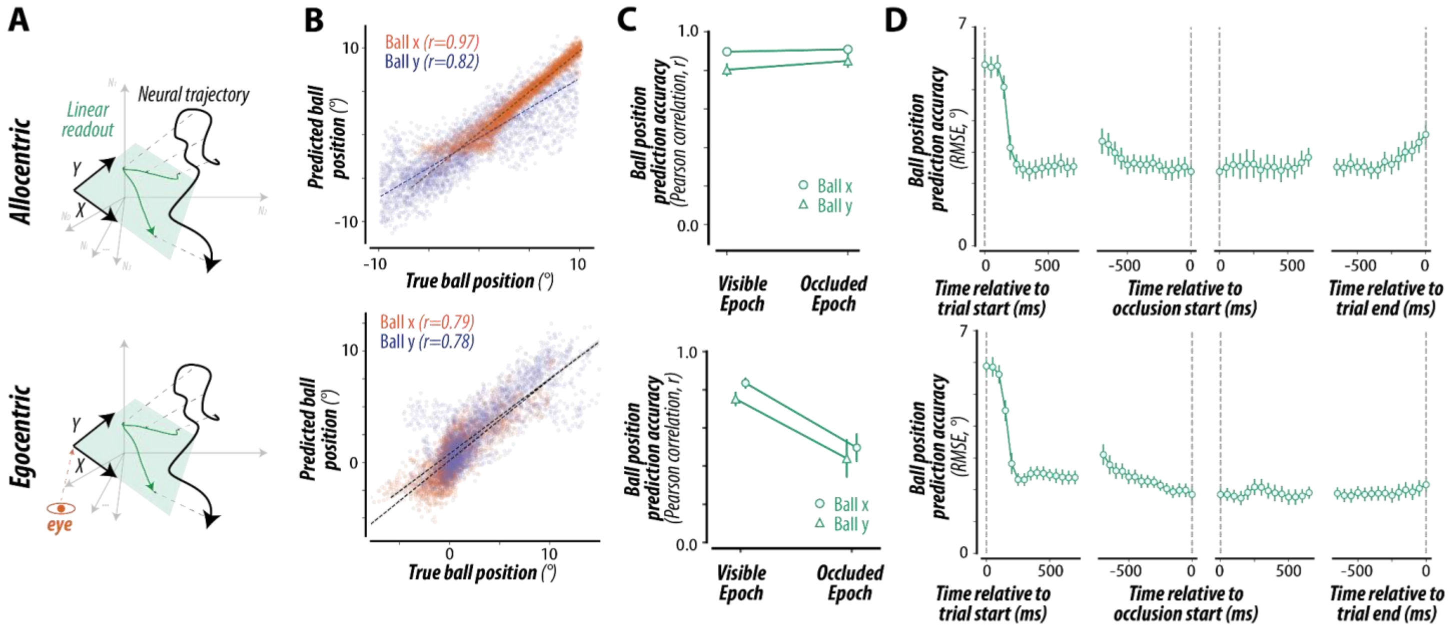

Figure S5. Population decoding analysis with allocentric (top row; same as Figure 3C-F) versus egocentric (bottom row) framework of reference. (A) Conceptual schematic (top: allocentric, bottom: egocentric). We used cross-validated linear regression to characterize the information content in the DMFC population related to the ball position. The only difference between the allocentric versus egocentric framework is that the ball positions are coded in the screen coordinate versus eye-centric coordinate, respectively (neural dynamics: black trace, predicted ball position: green trace, linear readout: green subspace). **(B)** Accuracy of DMFC readout (top: allocentric, bottom: egocentric). A linear readout from the DMFC population accurately captures the ball position, as shown by the scatters of true and predicted ball position along horizontal (ball x) and vertical (ball y) dimensions (r: Pearson correlation coefficient in the legend). Dotted lines correspond to least squares regression lines. **(C,D)** Dynamics of DMFC readout (top: allocentric, bottom: egocentric). The same linear readout can accurately capture the ball (x,y) position over the entire trial, i.e., (C) over both visible and occluded epochs, as quantified by a Pearson correlation, and (D) over 50 ms time-bins throughout the trial, as quantified by an Root-Mean-Squared-Error (RMSE) between true and predicted ball positions. Error bars denote mean±SEM. In the egocentric model (bottom), the occluded epoch showed lower prediction accuracy than the visible epoch ($p<10^{-30}$, $p<10^{-30}$ for x, y; Wilcoxon rank-sum test, N=100 test/test splits for cross validation for each x and y) but still significantly above zero ($p<10^{-15}$,$p<10^{-15}$; Wilcoxon signed-rank test, N=100 test/test splits for cross validation for each x and y). Source data are provided as a Source Data file.

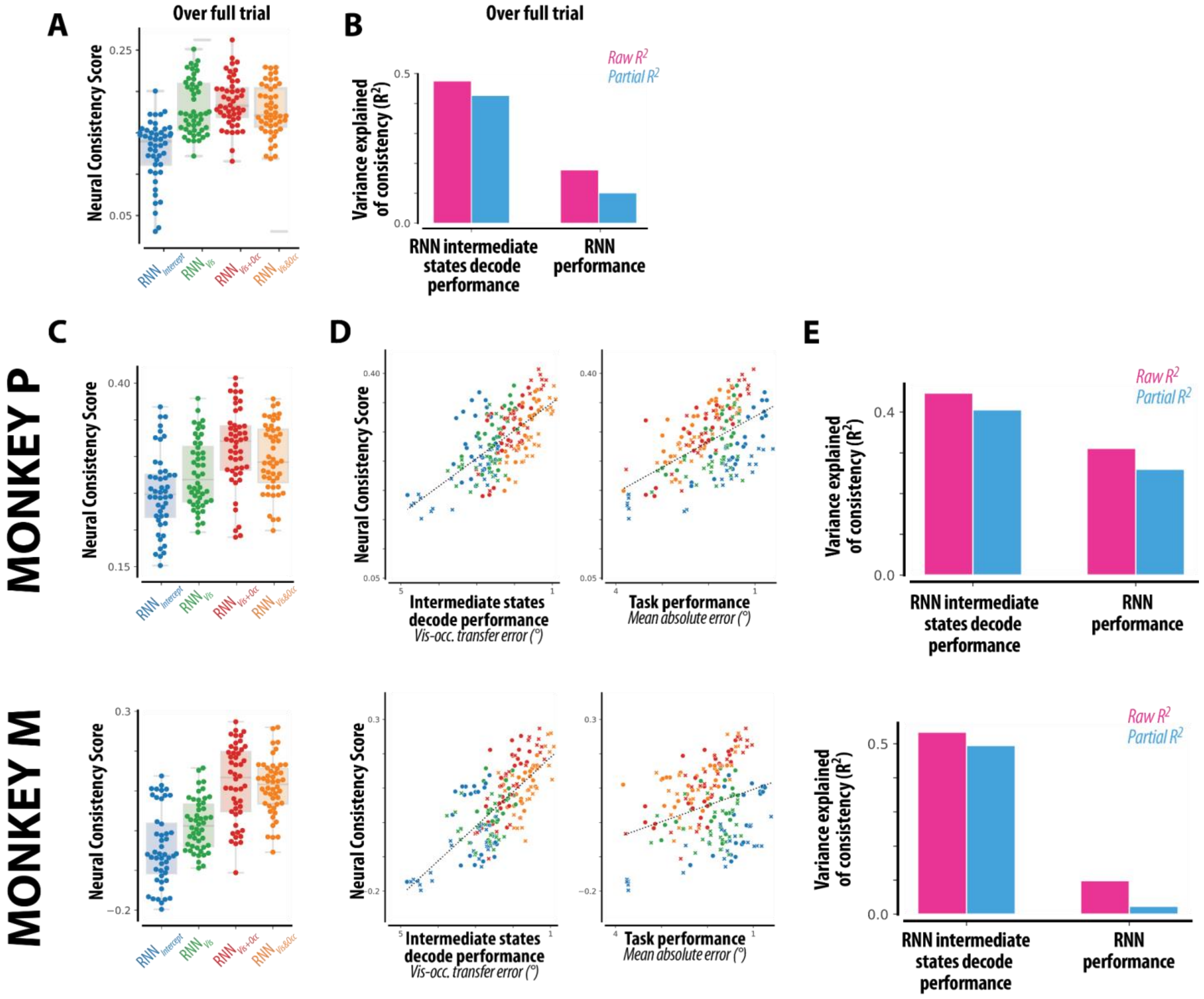

Figure S6. Complementary analyses for model-based test of the simulation hypothesis. (related to Figure 4). **(A,B)** Same as Fig. 4D,G in the main manuscript but when comparing RNNs and DMFC over the entire trial. The swarm plot shows individual models, and the boxplot shows the median, 1st and 3rd quartiles, and range of each distribution. **(B)** Quantifying the relationship shown in Figure 4D and 4E for the entire trial. The strength of dependence between functional attributes and consistency with DMFC dynamics is shown as a proportion of variance explained (R^2, pink bars). Partial R^2 (blue bars) measures this strength after accounting for covariations due to the other attribute. Across all RNNs, consistency with DMFC dynamics was well explained by intermediate state decode performance and also weakly explained by overall task performance. **(C, D, E)** Same information as Fig. 4D-G in the main manuscript, shown separately for the two animals. Source data are provided as a Source Data file.

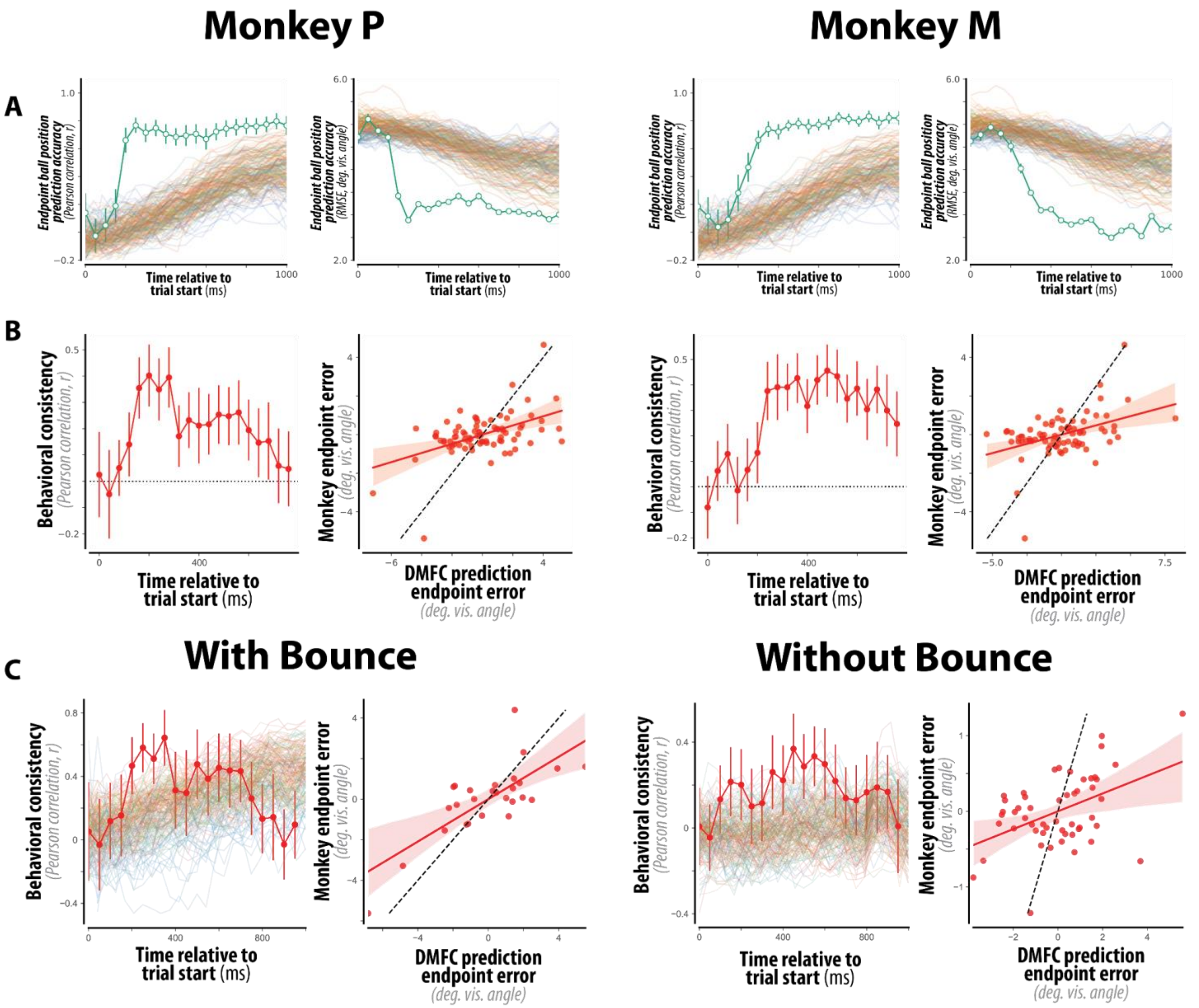

Figure S7. **Complementary analyses for rapid offline prediction in DMFC and RNN models.** (A,B) Same as Fig. 5B, 5C in the main manuscript, shown for the two animals separately. (C) Same as Fig. 5C in the main manuscript, shown for trials with and without bounces separately. Source data are provided as a Source Data file.

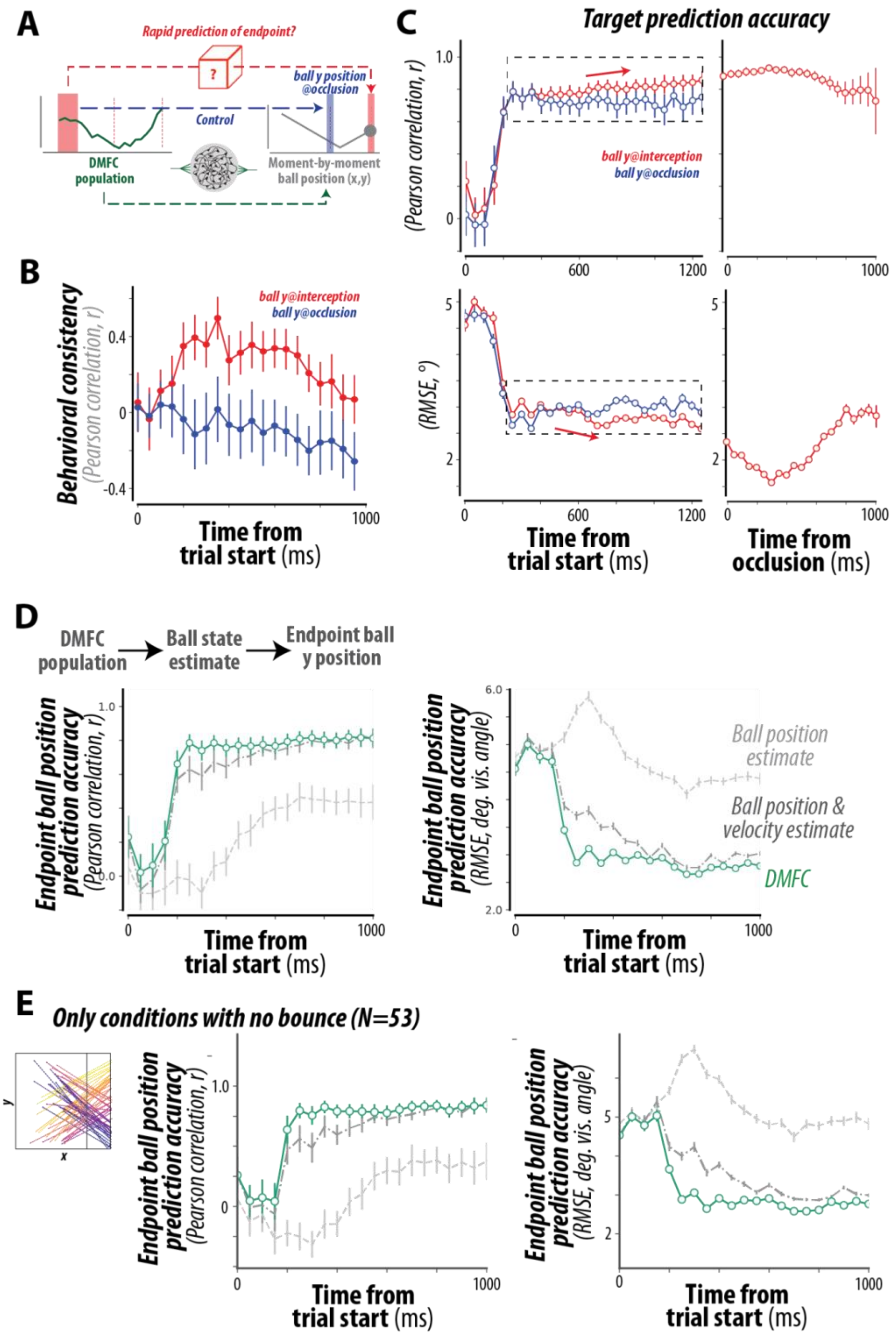

Figure S8. Complementary decoding analyses for offline prediction of the interception point. (A) Same as Fig. 5A with a schematic illustrating control analysis that attempted to decode the ball y-position at the time of occlusion start (middle blue; top red for rapid offline prediction, bottom green for online mental simulation processes). (B) Same as Fig. 5C but shown with control decoding result (blue). Different from the endpoint (red), DMFC's prediction for occlusion points was not correlated with the monkeys' behavioral errors. (C) Same as Fig. 5B but with the occlusion point decoding (blue). Both in Pearson correlation (top) and RMSE (bottom), prediction accuracy increases as a function of time for interception point (red, p<0.001, Wilcoxon rank-sum test; mean±SEM correlation with time: 0.57±0.029 for Pearson correlation, -0.35±0.037 for RMSE; dotted rectangles for window for computing the correlation with time; N=100 test/test splits for cross validation). For

the occlusion point, the correlation with time was much weaker (-0.12±0.039 for Pearson correlation; 0.26±0.042 for RMSE). (D) Comparison of DMFC representation of target with control hypothesis. Accuracy of different read-outs of the endpoint ball y-position (the target), quantified via Pearson correlation (left) and error (right). We first trained a read-out of the DMFC population to predict the moment-by-moment ball state. From this ball state estimate, we trained a read-out to predict the final target. The light gray line shows the accuracy of this target estimate, when the ball state corresponds to the ball position (x,y). The dark gray line shows the corresponding accuracy when the ball state corresponds to the ball position and velocity (x, y, dx, dy). The accuracy of the read-out of the target directly from the DMFC population (green) is greater than each of these controls ($p<0.001$ for all timepoints in [250ms, 500ms] for RMSE; two-sided permutation test; N=100 test/test splits for cross validation). (E) Same as panel D but only for conditions with no bounce (N=53 among 79 total conditions). Source data are provided as a Source Data file.
